# LEF1 reduces tumor progression and induces myodifferentiation in a subset of rhabdomyosarcoma

**DOI:** 10.18632/oncotarget.13887

**Published:** 2016-12-10

**Authors:** Julia Dräger, Katja Simon-Keller, Tobias Pukrop, Florian Klemm, Jörg Wilting, Carsten Sticht, Kai Dittmann, Matthias Schulz, Ivo Leuschner, Alexander Marx, Heidi Hahn

**Affiliations:** ^1^ Department of Human Genetics, University Medical Center, Göttingen 37073, Germany; ^2^ Institute of Pathology, University Medical Center Mannheim, Mannheim 68167, Germany; ^3^ Clinic for Internal Medicine III, Hematology and Medical Oncology, University Regensburg, Regensburg 93053, Germany; ^4^ Department of Hematology/Medical Oncology, University Medical Center Göttingen, Göttingen 37099, Germany; ^5^ Institute of Anatomy and Cell Biology, University Medical Center Göttingen, Göttingen 37075, Germany; ^6^ Center of Medical Research, Bioinformatic and Statistic, Medical Faculty Mannheim, Mannheim 68167, Germany; ^7^ Institute for Cellular and Molecular Immunology, University Medical Center Göttingen, Göttingen 37073, Germany; ^8^ Kiel Paediatric Tumor Registry, Department of Paediatric Pathology, University Hospital Schleswig-Holstein, Kiel 24105, Germany

**Keywords:** RMS, LEF1/TCF, WNT/β-catenin signaling

## Abstract

Rhabdomyosarcoma (RMS) is the most common soft tissue sarcoma in children and show characteristics of skeletal muscle differentiation. The two major RMS subtypes in children are alveolar (ARMS) and embryonal RMS (ERMS). We demonstrate that approximately 50% of ARMS and ERMS overexpress the LEF1/TCF transcription factor LEF1 when compared to normal skeletal muscle and that LEF1 can restrain aggressiveness especially of ARMS cells. LEF1 knockdown experiments in cell lines reveal that depending on the cellular context, LEF1 can induce pro-apoptotic signals. LEF1 can also suppress proliferation, migration and invasiveness of RMS cells both *in vitro* and *in vivo*. Furthermore, LEF1 can induce myodifferentiation of the tumor cells. This may involve regulation of other LEF1/TCF factors i.e. TCF1, whereas β-catenin activity plays a subordinate role. Together these data suggest that LEF1 rather has tumor suppressive functions and attenuates aggressiveness in a subset of RMS.

## INTRODUCTION

Rhabdomyosarcoma is an aggressive form of sarcoma that in the vast majority of cases occur in children younger than 18 years. Despite being a rare cancer, it accounts for approximately 40% of all soft tissue sarcomas in children [1, 2]. The two major subtypes in children are alveolar RMS (ARMS) and embryonal RMS (ERMS) showing different histological, genetic and clinical features. Thus, approximately 80% of ARMS show specific chromosomal translocations, which lead to the generation of PAX3-FOXO1 or PAX7-FOXO1 fusion proteins and are considered relevant in aetiology and prognosis [3]. Whereas fusion-positive ARMS are more aggressive, fusion-negative ARMS are clinically and molecularly similar to ERMS [4]. ERMS account for approximately two thirds of all RMS and are associated with a more favorable prognosis with a 5-year overall survival of approximately 73% compared to 48% for ARMS [1, 2, 5]. However, the survival rate for metastatic disease is only 40% for ERMS [6] and 10–30% for ARMS [7]. A better understanding of the molecular basis of this disease is important to improve current treatment schemes.

Activity of the canonical WNT (WNT/β-catenin) pathway is frequently involved in the development of tumors. Examples include colorectal cancer, malignant melanoma, medulloblastoma and several other tumor types [8]. Active canonical WNT signaling is indicated by elevated levels of β-catenin in the nucleus and/or cytoplasm. In the nucleus β-catenin interacts with the LEF1/TCF family of genes [8]. Like other TCFs, LEF1 is a transcription factor and is the prototypical mediator of WNT responses [9]. In the presence of WNT signals, LEF1 binds to β-catenin *via* its N-terminal β-catenin-binding domain and promotes context-dependent target gene transcription including *c-MYC* and *AXIN2*. Conversely, in the absence of the WNT signal, LEF1 represses WNT-responsive genes. WNT signaling can also be limited by dominant-negative LEF1 isoforms. These isoforms are produced by alternative promotors and lack the β-catenin-binding domain, thus preventing β-catenin access to targets (see reviews [10–12]).

Moreover, LEF1 and the other TCF family members are also known as architectural transcription factors, bend the DNA in a specific angle and exert functions independently of WNT/β-catenin signaling by e.g. interacting with cofactors such as ALY, Ets, TFE-3 and Sp1 [11]. Thus, LEF1 can promote growth of malignancies in the absence of β-catenin stabilization [13]. Moreover, LEF1 and TCF1 have intrinsic HDAC-activity, which is necessary for differentiation of CD8^+^ T-cells [14].

Because of these context dependent effects, LEF1 can function as either an oncogene or a tumor suppressor. For example, transplantation of LEF1-transduced bone marrow leads to acute myeloid leukemia and B-precursor ALL in the mouse [15]. Conversely, LEF1 can repress the transcription of *MYC* and thus act as a tumor suppressor in a subset of human T-ALL cases [16].

Although canonical WNT signaling plays an important role in muscle development [3] only few data on its role in RMS have been published. This is due to initial studies that revealed lack of nuclear β-catenin and lack of mutations in important components of the signaling pathway in RMS samples [17]. More recent papers now show that activation of canonical WNT signaling induces the expression of myogenic differentiation markers and inhibits proliferation of RMS cell lines [18, 19]. These data support a tumor-suppressive role of canonical WNT signaling in RMS that additionally promotes myogenic differentiation.

We here examined the role of LEF1 in RMS. Our experiments show that LEF1 can function as a tumor suppressor in this tumor entity and suggest that LEF1 is possibly one of the major mediators of RMS differentiation.

## RESULTS

### RMS biopsies express LEF1

**Figure 1:**
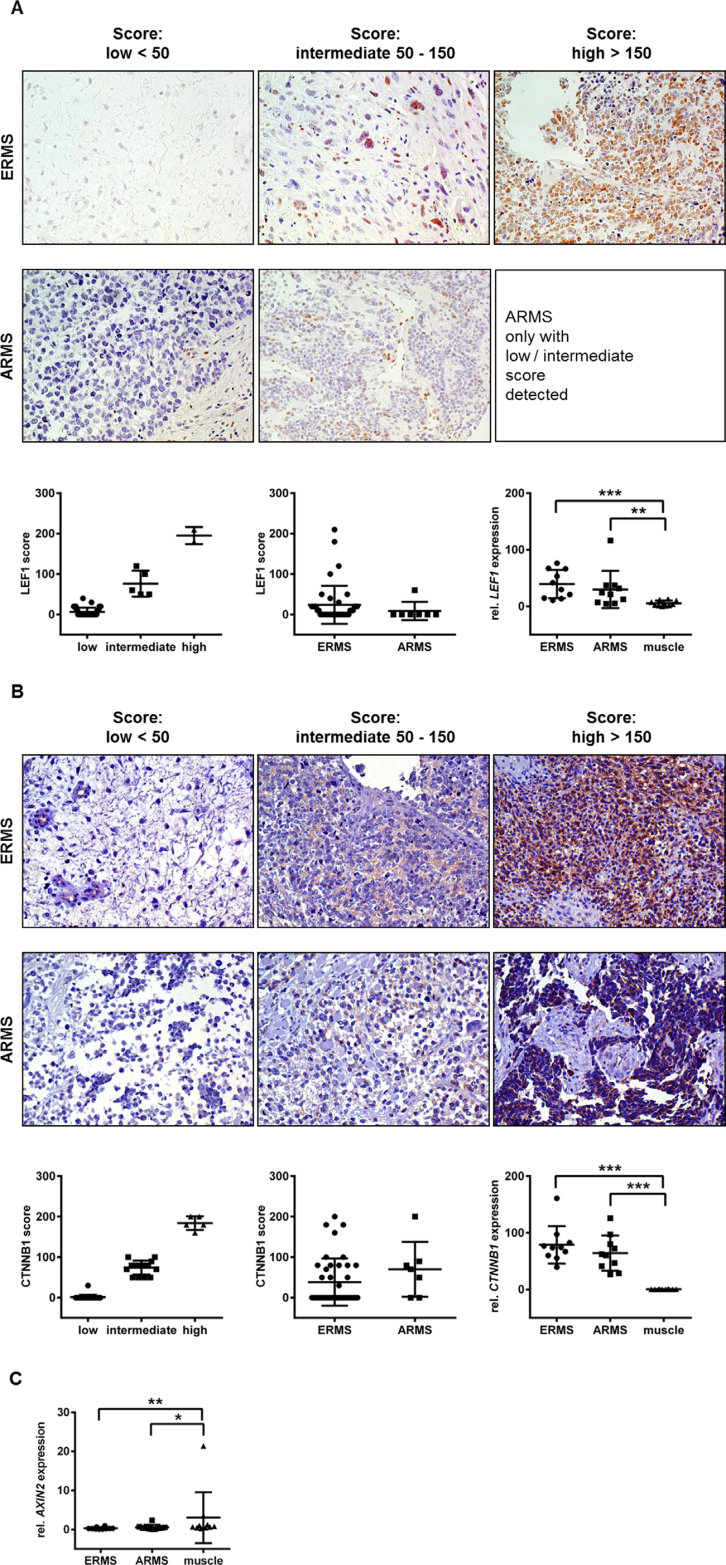
Immunohistochemical and/or qRT-PCR analyses of LEF1, β-catenin and AXIN2 in human ERMS and fusion-positive ARMS Representative data for LEF1 expression is shown in (**A**) and for β-catenin in (**B**). In each case upper panel shows immunohistochemistry stainings of the respective protein (LEF1 or β-catenin) in ERMS and fusion-positive ARMS. Results were scored by multiplying the percentage of positive cells by the intensity of the staining to subdivide studied samples into low, intermediate and high expressers. Lower left and center panels show the distribution of RMS in low, intermediate and high expressers according to the aforementioned scoring system and the distribution for ERMS and ARMS, respectively; right panels show *LEF1* (or *CTNNB1* in B) expression levels analyzed by qRT-PCR in fresh-frozen biopsies of human ERMS (*n* = 10) and fusion-positive ARMS (*n* = 10) compared to normal muscle (*n* = 10). (**C**) shows qRT-PCR analysis of *AXIN2* in the same biopsies. (A, B and C) Bars, 95% confidence intervals and mean values; ***P < 0.001, **P < 0.01, *P < 0.05 by Mann-Whitney *t*-test.

After quality control 41 ERMS and 7 fusion-positive ARMS samples arranged in a tumor microarray (TMA) were evaluable. The immunohistochemical analyses revealed that 43.1% of the RMS samples were positive for LEF1 although to a variable extend (Figure 1A, upper panel). When scoring the LEF1 positive samples (by multiplying the percentage of LEF1 positive cells by staining intensity) we found 41, 5 and 2 RMS with a low, intermediate and high score, respectively (Figure 1A, lower left panel). No ARMS with a high LEF1 score was detected and in general the LEF1 score was higher in ERMS compared to ARMS, however without reaching significance (Figure 1A, lower middle panel). LEF1 protein was exclusively found in the nucleus. Consistent but variable overexpression of *LEF1* was also seen on mRNA level in all fresh-frozen biopsies of our collection of 10 human ERMS and 10 human fusion-positive ARMS when compared to normal muscle (Figure 1A, lower right panel).

When β-catenin/*CTNNB1* expression was analyzed half of the RMS samples (47.1%) stained positive (Figure 1B, upper panel). Signals were detected in the cytoplasm with the exception of one ERMS case that also stained positive in the nucleus. Of the positive RMS, 28, 15 and 5 showed a low, intermediate or high β-catenin score, respectively (Figure 1B, lower left panel). Each β-catenin score was present in ERMS and ARMS (Figure 1B, upper panel and lower middle panel). On mRNA level all RMS expressed unequivocal high levels of this gene when compared to normal muscle (Figure 1B, lower right panel). We did not observe any correlation with LEF1/*LEF1* expression (data not shown).

Analysis of microarray-based expression data provided by Davicioni et al. [20] confirmed our findings. None of the performed comparisons between ARMS (PAX3-FOXO1) and ERMS as well as more detailed considerations between PAX3-FOXO1 translocation positive ARMS and various differentiation states of ERMS showed any significant difference between the two subtypes, nor correlation (Supplementary Table S1).

When the expression of the major downstream target of canonical WNT signaling *AXIN2* was analyzed, we found that this gene was rather downregulated in RMS compared to normal skeletal muscle (Figure 1C).

In summary, approximately half of ERMS and fusion-positive ARMS samples express LEF1 and β-catenin, with however variable and unrelated intensity. Furthermore, the common absence of nuclear β-catenin and of *AXIN2* expression suggests that canonical WNT signaling in general is not active in RMS. In this study, we tried to elucidate the role of LEF1, which can have functions independently of canonical WNT/β-catenin signaling (see introduction), in RMS.

### Establishment of LEF1 knockdown RMS cell lines

**Figure 2:**
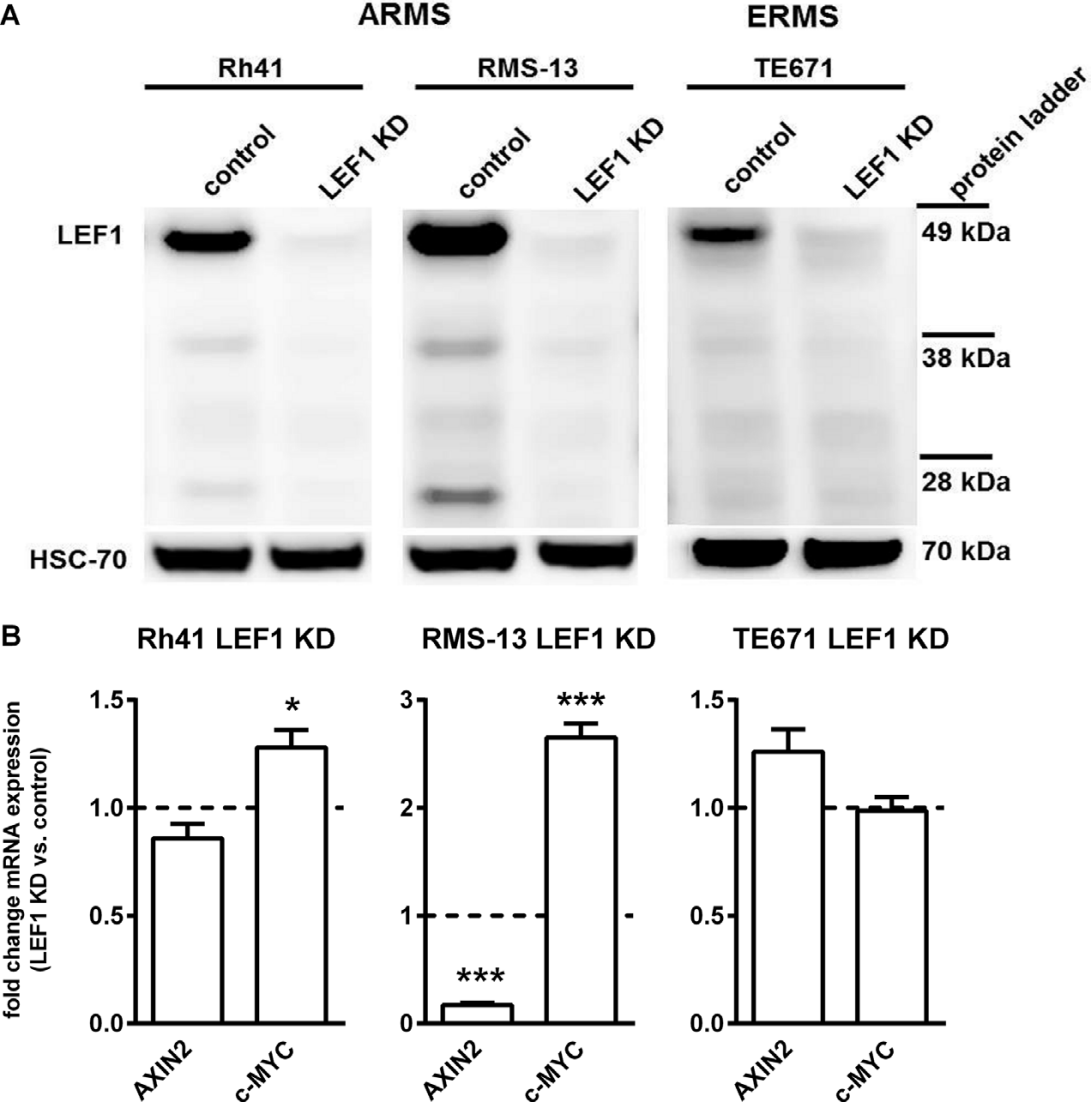
Generation of stable LEF1 knockdown (LEF1 KD) RMS cell lines and expression analysis of WNT target genes (**A**) Representative LEF1 Western blot of human ARMS cell lines Rh41 and RMS-13 and the human ERMS cell line TE671 stably expressing a LEF1 shRNA (LEF1 KD) or empty vector control (control). HSC-70 served as loading control. Protein ladder is shown for estimation of protein size. (**B**) Expression of *AXIN2* and c-*MYC* in Rh41 LEF1 KD, RMS-13 LEF1 KD and TE671 LEF1 KD are shown as fold expression to the respective control cells that were set to 1 (dashed line). Gene expression levels were normalized to *18S* rRNA expression levels. Data represent mean+SEM of at least three independent experiments performed in duplicates and measured in triplicates; *P < 0.05, ***P < 0.001 by Students *t*-test.

In order to analyze the function of LEF1 in RMS, we sought to either overexpress or delete LEF1 in LEF1 negative or LEF1 positive RMS cell lines, respectively. Since all examined cell lines expressed LEF1 (Supplementary Figure S1), the effects of a LEF1 knockdown (LEF1 KD) in the ERMS cell line TE671 and the ARMS cell lines Rh41 and RMS-13 were studied. Since these RMS cell lines express the full-length LEF1 isoform of 44 kDa, whereas the truncated isoform lacking the β-catenin binding site (31 kDa and 23 kDa; see [9, 21]) were barely detected (Figure 2A), we conclude that these cell lines express functional LEF1 that can interact with β-catenin. In addition, all cell lines express the downstream targets of canonical WNT signaling, *AXIN2* and *c-MYC* (Supplementary Figure S2). A stable LEF1 KD in the cell lines (Figure 2A) resulted in a significant downregulation of the LEF1 target *AXIN2* only in RMS-13 LEF1 KD cells (Figure 2B). Interestingly, the LEF1 target *c-MYC* was significantly increased in RMS-13 LEF1 KD and Rh41 LEF1 KD cells (Figure 2B). This is similar to T-cell acute lymphoblastic leukemia, in which LEF1-inactivated cases show increased levels of *MYC* expression when compared with cases with intact LEF1 [16]. No significant effects of the LEF1 KD on the expression of the two mentioned genes were seen in TE671 cells.

### LEF1 plays a subsidiary role for canonical WNT signaling activity in RMS

We next investigated whether LEF1 is important for the maintenance of canonical WNT signaling activity in RMS cells.

**Figure 3:**
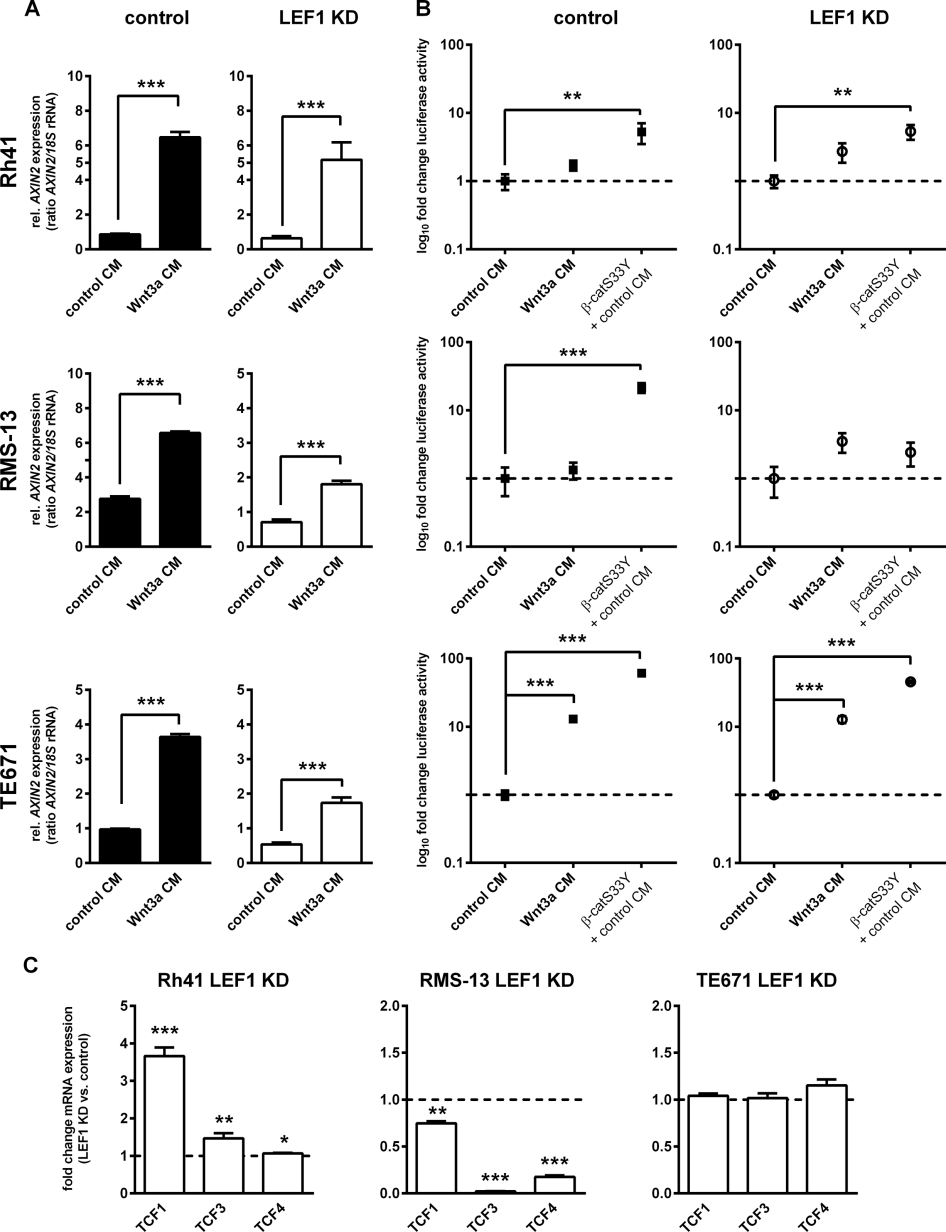
LEF1-dependent modulation of canonical WNT signaling activity in RMS cell lines (**A**) Expression of *AXIN2* in Rh41 LEF1 KD, RMS-13 LEF1 KD and TE671 LEF1 KD and respective control cells in response to Wnt3a conditioned medium (Wnt3a CM) or control medium (control CM). Gene expression levels were normalized to *18S* rRNA expression levels. Data represent mean+SEM of at least two independent experiments performed in duplicates and measured in triplicates. (**B**) To analyze β-catenin-dependent WNT signaling in response to LEF1 KD, cells were transfected with *SuperTOPFlash* (TOP) containing multiple TCF/LEF-binding sites and *Renilla* reporter plasmid for normalization. Luciferase activity was measured 5 days after transfection in response to Wnt3a or control CM. Transfection of the cells with pCl-neo-β-catS33Y (β-catS33Y) served as positive control. Data show the 95% confidence intervals of at least two independent experiments performed in duplicates and are depicted as fold luciferase activity to cells treated with control CM (set to 1; dashed line). (**C**) Expression of *TCF1, 3* and *4* in Rh41 LEF1 KD, RMS-13 LEF1 KD and TE671 LEF1 KD are shown as fold expression to the respective control cells that were set to 1 (dashed line). Gene expression levels were normalized to *GAPDH* expression levels. Data represent mean + SEM of at least four independent experiments measured in triplicates. (A, B and C) *P < 0.05, **P < 0.01, ***P < 0.001 by Students *t*-test.

First, we transfected RMS LEF1 KD and respective control cells with the *SuperTOPFlash* (TOP) plasmid containing multiple TCF/LEF-binding sites or its negative control vector *SuperFOPFlash* (FOP) along with *Renilla* reporter plasmid for normalization. In order to activate canonical WNT/β-catenin signaling, the cells were incubated with Wnt3a containing medium (Wnt3a CM). All other cells were maintained in control conditioned medium (control CM). Co-transfection with the pCl-neo-β-catS33Y plasmid expressing a stabilized and active β-catenin (β-catS33Y) served as a positive control (please note that control experiments employing *SuperFOPFlash* are shown in Supplementary Figure S3). As shown in Figure 3A, all cell lines significantly upregulated *AXIN2* mRNA levels in response to Wnt3a CM (48 h incubation; Figure 3A). However, the TOP reporter was not significantly activated by Wnt3a in the ARMS cell lines Rh41 and RMS-13 irrespective of the LEF1 deletion (Figure 3B), although transfection with activated β-catenin (β-catS33Y) revealed strong luciferase induction except in RMS-13 LEF1 KD cells (Figure 3B). In contrast, the ERMS cell line TE671 was responsive to Wnt3a CM treatment and showed a more than 10-fold TOP induction (Figure 3B). Because the induction was of similar magnitude in both TE671 control and TE671 LEF1 KD cells (see Supplementary Figure S4) the data indicate that LEF1 is not necessary for activation of the TOP reporter, i.e. it is dispensable for canonical WNT/β-catenin-dependent signaling.

In ARMS cell lines *AXIN2* expression was induced by Wnt3a despite lack of TOP reporter activity. Thus, the expression of *AXIN2* must be regulated by other factors than β-catenin, e.g. by E2F1 [22]. The lack of TOP reporter activity in ARMS cells also suggests that the parental RMS-13 and Rh41 cells may i) have a mutation in the endogenous β-catenin that prevents induction of Wnt3a-mediated signaling activity or ii) possess a mechanism that prevents endogenous β-catenin from binding to the reporter plasmid e.g. it is possible that β-catenin is retained in the plasma membrane or cytoplasm (for review see [23]). Finally, the fact that β-catS33Y did not induce signaling activity in RMS-13 LEF1 KD cells additionally argued for a factor that prevents β-catS33Y from binding to the TCF-binding site of the reporter plasmid after LEF1 depletion.

To answer these questions, we first sequenced the β-catenin coding region (see Supplementary Table S3 for sequencing primers). Because β-catenin in the analyzed cell lines was not mutated (data not shown), we next analyzed whether β-catenin was able to translocate to the nucleus after stimulation with Wnt3a. Immunofluorescent staining demonstrated predominant nuclear accumulation of β-catenin in the ERMS cell line TE671 (Supplementary Figure S5). In contrast, nuclear β-catenin was never detected in RMS-13 cells, whereas a very weak but distinct nuclear β-catenin staining was detected after incubation with Wnt3a CM in approximately 10% Rh41 cells. This is similar to the results of the TOP/FOP reporter assay that showed a 1.3-fold and 1.7-fold induction of TOP activity in RMS-13 and Rh41 control cells after Wnt3a treatment, respectively (*P* = 0.58 and *P* = 0.085; see Figure 3A). Thus, these results are in favor of the hypothesis that endogenous β-catenin in Rh41 and RMS-13 is rather hold back in the plasma membrane or cytoplasm.

Finally, we examined the expression of other LEF1/TCF factors that also interact with β-catenin and generally can activate (TCF1 and TCF4) or inhibit (TCF3 and TCF4) canonical WNT signaling [24, 25]. All cell lines expressed *TCF1*, *3* and *4*, however to a variable extend (Supplementary Figure S6). When the influence of the LEF1 KD on the expression of the *TCFs* was examined, we found that all factors were significantly upregulated in Rh41 LEF1 KD cells, whereas all 3 factors were downregulated in RMS-13 LEF1 KD (Figure 3C). In TE671 cells, we did not find any significant changes. Together, these data suggest that the lack of TOP activation in β-catS33Y-transfected RMS-13 LEF1 KD cells may be due to extreme downregulation of all TCFs upon LEF1 depletion. However, this speculation remains to be verified in the future.

### LEF1 can antagonize aggressiveness of RMS

**Figure 4:**
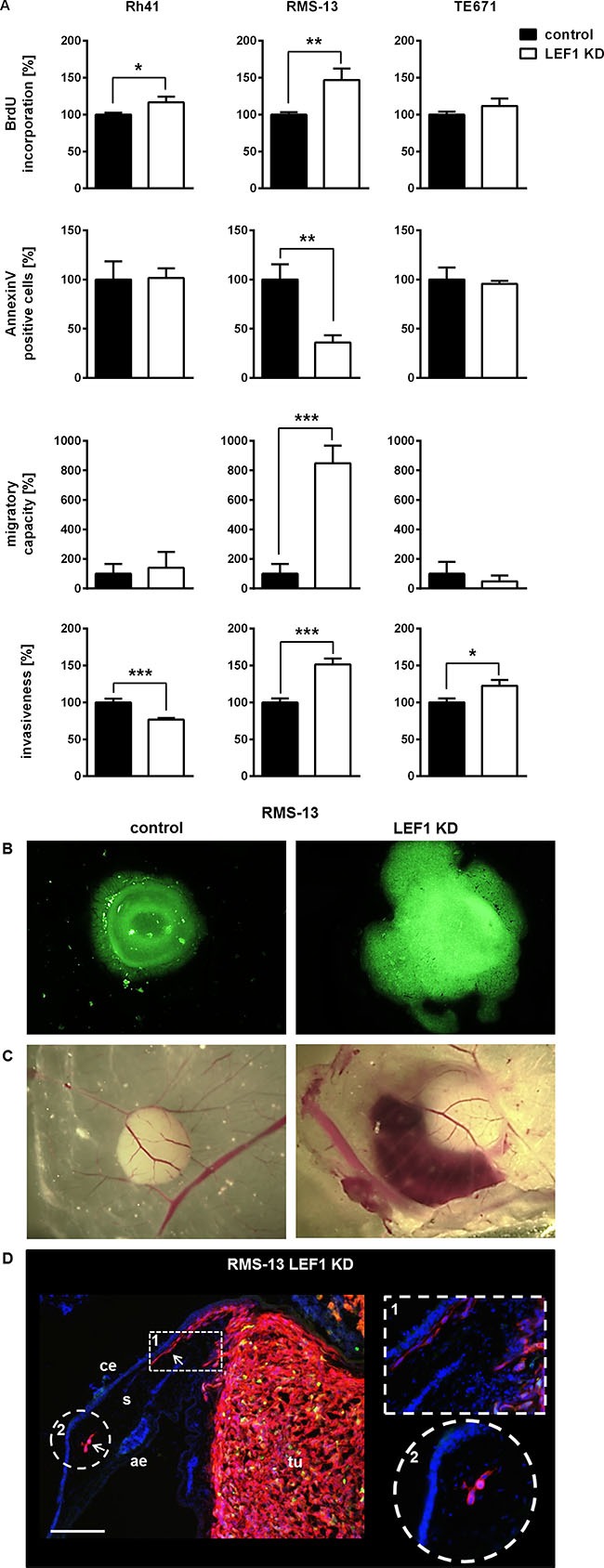
LEF1-dependent regulation of proliferation, apoptosis, migration and invasiveness of RMS cell lines (**A**) Proliferation, apoptosis, migratory capacity and invasiveness of the cells were analyzed by BrdU incorporation assay, FACS, trans-well migration and Boyden chamber assay, respectively. Data represent mean+SEM of at least two independent experiments performed in triplicates (BrdU incorporation assay, migration assay for RMS-13) or duplicates (apoptosis, migration and invasion assay). For all measurements the respective values from control cell lines were set to 100%. Comparisons were made with Students *t*-test; *P < 0.05, **P < 0.01, ***P < 0.001. (**B** and **C**) shows a representative intravital imaging of RMS-13 LEF1 KD and control cells in the CAM model at day 3 and 7 in each cohort post inoculation, respectively. (B) Due to stable transduction with lentiviral pGIPZ vector that expresses GFP the growth of the cells could be visualized by fluorescence (20-fold magnification). (C) RMS-13 LEF1 KD tumor growth is accompanied by destruction of vessels and hemorrhage (10-fold magnification). (**D**) Immunofluorescence staining of cryosection of tumors derived from RMS-13 LEF1 KD cells with anti-HLA-A,B,C (red) and DAPI (blue). White arrows mark tumor cells invading the stroma (s), and are also shown in the insets. Depicted are the chorion epithelium (ce), the allantoic epithelium (ae) and the tumor (tu). Scale bar 70 μm.

Next, the RMS LEF1 KD cell lines were analyzed with respect to proliferation, apoptosis and their migratory and invasive properties. As shown in Figure 4A, the LEF1 KD increased the proliferative capacity of all three cell lines. Compared to the control-transduced cell lines the difference was significant for Rh41 LEF1 KD and RMS-13 LEF1 KD. In the latter cell line we also observed a significant decrease in the number of apoptotic cells and a significant increase in the number of cells that migrated through the membrane insert or invaded the Matrigel in the Boyden chamber invasion assay (Figure 4A). In contrast to RMS-13, the LEF1 KD in Rh41 cells resulted in a significant decrease of the invasive capacity when compared to control cells. In TE671 cells, the LEF1 KD led to a significant increase of cell invasiveness.

Despite the fact that i) the response of the used RMS cell lines to the LEF1 KD is heterogeneous and ii) the LEF1 KD decreases the invasive capacity of Rh41 cells, the data show that LEF1 depletion generally results in increased RMS proliferation and can increase the migratory/invasive properties of RMS cells. It also can inhibit apoptosis. In summary, the presence of LEF1 can attenuate the aggressiveness of RMS cell lines. This was confirmed *in vivo* using the chicken CAM model, which is an established assay method for tumor growth and invasiveness. To this end, RMS-13 control and RMS-13 LEF KD cells were seeded on the CAM of chicken embryos and were allowed to form tumors until day 7. In agreement with the *in vitro* experiments, the LEF1 KD in RMS-13 cells augmented tumor growth. Thus, 3 days after inoculation, the LEF1 KD cells have formed larger tumors in comparison to the control (Figure 4B). 7 days after inoculation, the LEF1 KD tumors showed hemorrhage, which was never observed in the controls (Figure 4C). Additionally, HLA immunofluorescence staining detected human MHC-identified RMS-13 LEF1 KD cells in the stroma of the CAM (Figure 4D), confirming that the LEF1 KD increased the migratory and invasive properties of RMS-13 cells.

### LEF1 can contribute to myogenic differentiation in RMS

**Figure 5:**
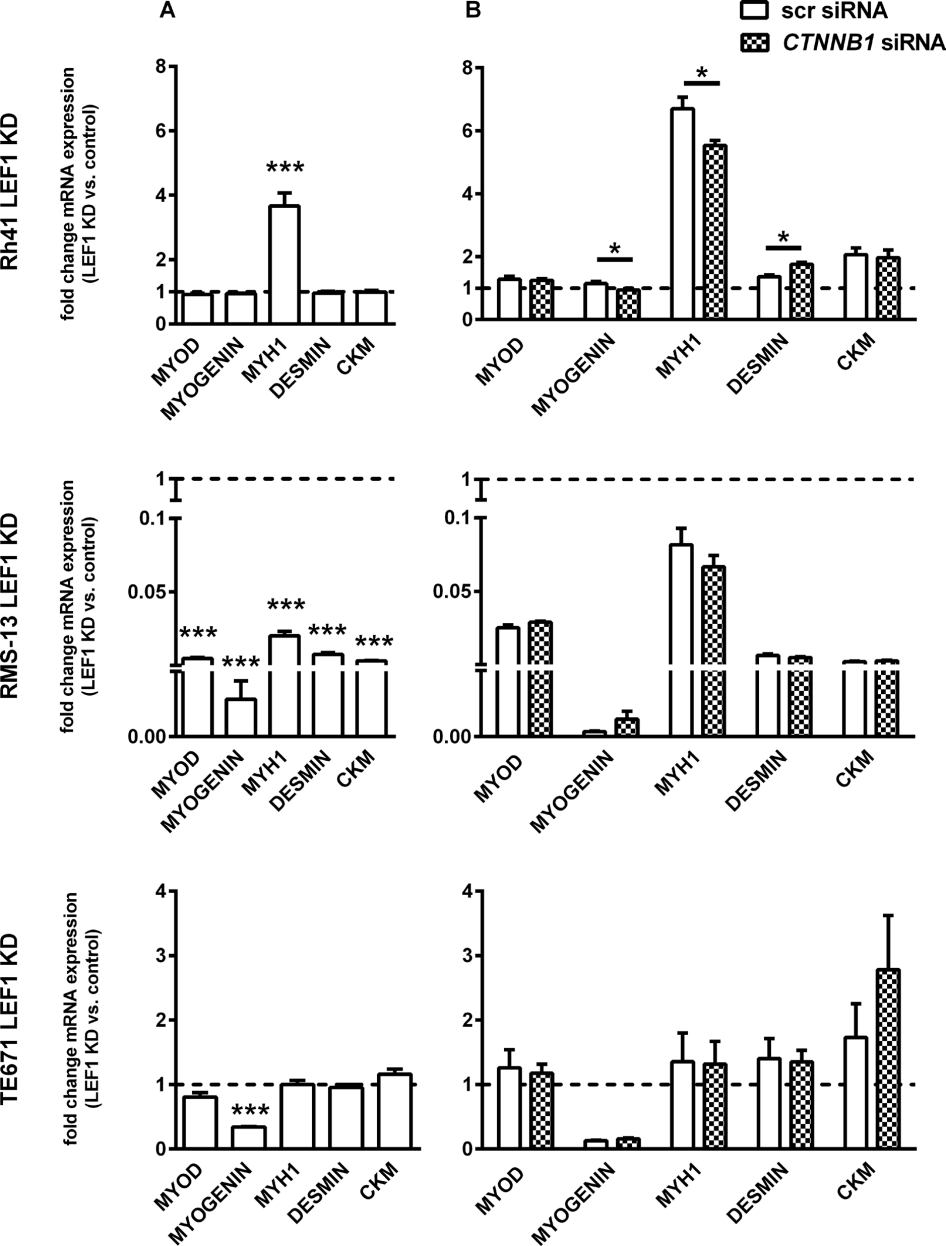
LEF1-dependent expression of muscle differentiation markers in RMS cell lines (**A**) Expression of *MYOD, MYOGENIN, MYH1, DESMIN* and *CKM* in Rh41 LEF1 KD, RMS-13 LEF1 KD and TE671 LEF1 KD are shown as fold expression to the respective control cells that were set to 1 (dashed line). (**B**) Expression of the same markers in the same cells after transfection with scrambled siRNA (scr siRNA) or *CTNNB1* siRNA. Significances are shown for values after transfection with scr siRNA versus *CTNNB1* siRNA. (A and B) Gene expression levels were normalized to *18S* rRNA expression levels. Data represent mean + SEM of at least two independent experiments performed in duplicates and measured in triplicates; *P< 0.05, ***P < 0.001 by Students *t*-test.

Since promotion of myodifferentiation can be tumor suppressive in RMS development [26], we also investigated whether LEF1 is involved in muscle differentiation processes. Indeed, in RMS-13 cells, LEF1 depletion was accompanied by an almost complete transcriptional suppression of the differentiation markers *MYOD*, *MYOGENIN*, *MYH1*, *DESMIN* and *CKM* (Figure 5A). This was different in Rh41 and TE671 cells, in which the LEF1 KD significantly increased *MYH1* and decreased *MYOGENIN* mRNA level, respectively, but did not result in other significant changes (Figure 5A). Together, these data demonstrate that in dependency on the cellular context, LEF1 can induce myodifferentiation in RMS.

Since recent papers proposed a very important role of β-catenin in myogenic differentiation of RMS cells [18, 19], we finally investigated if β-catenin was essential for the role of LEF1 in myodifferentiation. For this purpose, all cell lines were transiently transfected with *CTNNB1* specific siRNA. The successful knockdown was verified by qRT-PCR and Western Blot (see Supplementary Figure S7). Except a significant up- or downregulation of *DESMIN* or *MYH1* and *MYOGENIN*, respectively, in Rh41 LEF1 KD cells (Figure 5B), the *CTNNB1* KD itself did not change the expression of muscle markers. Together these data suggest that LEF1 is one of the main regulators of myodifferentiation in RMS and that β-catenin plays an inferior role in this process.

## DISCUSSION

We here show that both ERMS and ARMS can express LEF1. Despite high heterogeneity among patient samples and cell lines, our LEF1 knockdown experiments using Rh41, RMS-13 (ARMS) and TE671 (ERMS) cell lines demonstrate that LEF1 can reduce tumor progression and induce myodifferentiation.

LEF1 is an important interaction partner of activated β-catenin in the nucleus. In addition, it can be a downstream target of β-catenin (see introduction and [11]). However, nuclear β-catenin signals were detected in only one ERMS sample of the TMA, which is in line with previously published data [19]. The general absence of nuclear β-catenin in RMS tissue may also explain i) the low levels of the widely recognized target of WNT/β-catenin signaling *AXIN2* in clinical samples (see Figure 1C) and ii) the lack of correlation between β-catenin/*CTNNB1* and LEF1/*LEF1* expression levels. It also may indicate that WNT/β-catenin signaling is inactive in most RMS. Indeed, our cell culture experiments using Wnt3a suggest that activation of WNT/β-catenin signaling is only possible in subsets of RMS, such as ERMS subtypes. In addition, the fact that *AXIN2* activation was not seen in primary samples but in all cell lines after treatment with Wnt3a argues for a subordinate role of this WNT ligand in the primary samples.

Overexpression of LEF1 has been detected in many tumor entities. Although LEF1 frequently acts as an oncogene (for review see [27]), it also can function as a tumor suppressor gene [16]. In RMS cells, LEF1 apparently mainly acts as a suppressor. Thus, LEF1 generally attenuates proliferation of RMS cell lines, which was significant for the ARMS cell lines RMS-13 and Rh41. Furthermore, LEF1 antagonizes invasiveness of RMS-13 and TE671 that goes along with inhibition of migration and induction of apoptosis in RMS-13 cells. It apparently can also trigger apoptotic processes in RMS-13 cells (Figure 4A), as has been described for colorectal cancer [28]. This suggests that LEF1 can counteract the aggressiveness of RMS. However, the fact that LEF1 fosters invasiveness of Rh41 cells indicates that this tumor suppressive function may be restrained to specific RMS subgroups.

The anti-proliferative effect of LEF1 in RMS may be related to attenuation of c-MYC expression. Thus, the LEF1 KD in the ARMS cell lines Rh41 and RMS-13 results in significant upregulation of *c-MYC* expression and cellular proliferation. This is similar to T-ALL, in which LEF1 inactivation increases expression of *MYC* and MYC targets [16]. Because c-MYC drives proliferation of many tumor cells including RMS [29, 30], it is tempting to speculate that the increased *c-MYC* levels in ARMS LEF1 KD cells are responsible for induction of proliferation.

Furthermore, it is possible that TCF1, TCF3 and TCF4 are associated with the LEF1-mediated RMS phenotype. This is first illustrated by the effects of the LEF1 KD on invasiveness. While LEF1 KD in RMS-13 induces invasiveness, the opposite is the case for Rh41 cells where invasion is reduced. This was accompanied by a down- or upregulation of all TCFs, respectively. In TE671, neither TCF levels nor invasive capacity were affected (Figures 3C and 4A). This indicates that the dosage and/or composition of LEF1/TCFs factors may regulate the invasiveness of RMS cells.

Secondly, downregulation of all TCFs in RMS-13 cells upon *LEF1* deletion could also explain downregulation of *AXIN2*, which was not seen in the other cell lines.

Thirdly, TCFs may also play a role in myogenic differentiation (and thus aggressiveness) modulated by LEF1, at least in ARMS cell lines. Normal myogenic differentiation leads to the formation of myotubes. Neoplastic differentiation in RMS mostly follows the same pathway, but with less efficient tube formation and the overall proliferative and tumorigenic capacity of rhabdomyosarcoma is inversely related to the degree of myogenic differentiation [5, 31, 32]. Indeed, in RMS-13 cells LEF1 is necessary for the induction of both early (*MYOD*) and late (*MYOGENIN, MYH1*, *DESMIN* and *CKM*; [33]) muscle differentiation markers (see Figure 5A). Also in TE671 cells it is necessary for *MYOGENIN* expression. In contrast in Rh41 cells, LEF1 apparently suppresses the late marker *MYH1*, whereas the expression of other markers is not influenced. In principle, late differentiation markers like *MYH1* are induced after cell cycle arrest [34]. However, the increase in BrdU incorporation in Rh41 LEF1 KD cells (see Figure 3A) strongly argues against this scenario. In addition, our setting rather implicates a β-catenin-independent, LEF1-mediated regulation of myodifferentiation. Indeed, β-catenin-independent LEF1 functions are well known and examples encompass interaction with ATF2 factors [13] and with the intracellular domain of Notch [35]. Finally, LEF1 together with TCF1 has intrinsic HDAC activity that recently was shown to repress genes counteracting cellular differentiation in specific contexts [14]. In our settings, LEF1 induces the expression of TCF1 and of muscle lineage markers in RMS-13 cells. This is different in Rh41 cells, in which LEF1 rather suppresses TCF1 and myogenic differentiation. Therefore it is tempting to speculate that there are subgroups of RMS, in which differentiation (and concomitantly aggressiveness) is epigenetically regulated by LEF1 and TCF1. Indeed, a recent study divides RMS into 4 molecular subtypes based on their genetic and epigenetic signature [36]. Whether these speculations are true or not remains to be established in the future.

Regarding myogenic differentiation of RMS cell lines our data contrasts with recent studies implicating β-catenin-driven canonical WNT signaling in myogenic differentiation of the alveolar RMS cell lines Rh4 and Rh30 and the embryonal lines RD and RD18 [19]. RMS-13 is sometimes thought to be related to Rh30 and perhaps derived from the same patient tumor. The same applies to Rh41 and Rh4 [37]. Nevertheless, the origin of RMS-13 and Rh30 is not clear, and Rh41 and Rh4 were developed in two different laboratories [37]. Therefore, the differences between the studies may reflect heterogeneity of different RMS cell clones. However, it is also possible that the observed differences between the cell lines depend on the histology of the individual tumor part that has been used for establishment of the individual cell line. Regardless of whether these assumptions are correct, our data indicate that the recently proposed new treatment option for RMS using GSK3 inhibitors to activate β-catenin-driven WNT signaling [38] may be only of benefit for specific subtypes of RMS, but not for others in which this pathway does not play a role.

## MATERIALS AND METHODS

### Biopsies

A tumor microarray (TMA) with 125 RMS biopsies from the Pediatric Tumor Register, Kiel, Germany and 20 RNA samples from the CWS (“Cooperative Weichteilsarkom Studiengruppe”) tissue bank, Stuttgart, Germany (S1–S20) were studied. Histopathology of all cases was centrally reviewed by Prof. I. Leuschner (Pediatric Tumor Registry, Kiel, Germany). All patients were treated according to CWS protocols. All studies were approved by the appropriate ethics and review committees. Written informed consent according to the Declaration of Helsinki was obtained from all patients or their legal guardians, depending on the patients’ age.

### Microarray analysis

Expression of *LEF1* and *CTNNB1* was also evaluated in a publicly available RMS microarray data set [20] (available at ftp://caftpd.nci.nih.gov/pub/caARRAY/experiments/caArray_trich-00099/,). A Custom CDF Version 20 with ENTREZ based gene definitions was used to annotate the arrays. The Raw fluorescence intensity values were normalized applying quantile normalization and RMS background correction. An ANOVA was performed to identify differential expressed genes using a commercial software package SAS JMP10 Genomics, version 6, from SAS (SAS Institute, Cary, NC, USA). A false positive rate of a = 0.05 with FDR correction was taken as the level of significance.

### Immunohistochemistry

The TMA, consisting of 25 ARMS (22 fusion-positive, 3 fusion-negative) and 100 ERMS samples, were sectioned at 5 μm for histological analyses. Hematoxylin eosin (H&E) staining was performed by standard methods. The paraffin sections were stained using a rabbit monoclonal anti-LEF1 antibody (clone EPR2029Y, 1:250, pH 9.0 from Abgent; detects the 44 kDa full-length and the 31 kDa and 23 kDa isoforms) and a mouse monoclonal anti-β-catenin antibody (clone 5H10, 1:200, pH 6.0 from Zymed). Immunohistochemistry was performed as described in detail elsewhere [39] using the following chemicals and reagents: antigen retrieval in Novocastra antigen retrieval solution pH 6.0 or pH 9.0 (Leica, Wetzlar, Germany); blocking of endogenous peroxidase (DAKO blocking solution, DAKO) and detection of bound antibodies by the immunoperoxidase/DAB-based DAKO REAL detection system (DAKO).

The proportion of LEF1- and β-catenin-positive cells as well as the intensity of the staining was estimated by a pathologist (weak staining 1; moderate staining 2, strong staining 3). Results were scored by multiplying the percentage of positive cells by the intensity [40].

Immunofluorescence staining of cryosections (CAM assay) was performed after incubation for 1 h with blocking reagent (PBS, 1% BSA). Sections were incubated overnight (ON) with an anti-HLA-A,B,C antibody followed by staining for 1 h with secondary Alexa Fluor 594-labeled antibody diluted in antibody solution mixed with DAPI (1:10,000). After every step specimens were rinsed twice with PBS. Samples were mounted with Fluoromount-G (Sigma-Aldrich Chemistry GmbH) and dried ON at room temperature (RT). Stained specimens were studied with Zeiss Axio Imager. Z1 (Carl Zeiss Goettingen) and filter sets 38HE, 43 and 49. Used primary antibodies and corresponding secondary antibodies are shown in Supplementary Table S2.

For immunofluorescence staining cells were grown on slides and were fixed with 2% paraformaldehyde followed by methanol at RT for 10 min or −20°C for 5 min, respectively. After washing with PBS, cells were permeabilized with 0.5% Triton X-100 (in PBS) for 5 min at RT and unspecific antigens were blocked with 4% BSA (in PBS) for 1 h in a moist chamber. Then the slides were rinsed twice with PBS and the cells were stained with anti-β-catenin antibody ON at 4°C followed by incubation with TRITC-conjugated anti-mouse as secondary antibody for 1 h at RT. Finally, cells were mounted with ProLong Gold antifade reagent with DAPI (Thermo Fisher Scientific) and analyzed by fluorescence microscopy (Olympus BX60, equipped with U-RLF-T). Serial pictures at 60-fold magnification were taken for each chamber and fluorescence images were acquired by using a Color View camera (Soft Imaging System) and the software CellSens (Olympus Life Science). Two independent experiments were performed. Used primary antibodies and corresponding secondary antibodies are shown in Supplementary Table S2.

### Real time quantitative PCR

Total RNA was extracted using TRIzol reagent (Thermo Fisher Scientific). cDNA was synthesized using Superscript II and random hexamers (Invitrogen) or using the “RevertAidTM H Minus First Strand cDNA Synthesis Kit” (Thermo Fisher Scientific). Gene expression was quantified by SYBR Green-based qRT-PCR assays on the “Step one plus system” or by the ABI Prism HT 7900 Detection System instrument and software (Applied Biosystems). Data were analyzed by using the GraphPad Prism software tool (San Diego, CA, USA) or by the standard curve method for relative quantification, respectively. The primers for amplification of target transcripts are shown in Supplementary Table S3.

All primer pairs were intron-flanking, except of the primers for *18S* and *MYOD*. Amplification of *18S* rRNA or *GAPDH* mRNA served to normalize the amount of sample cDNA. Gene expression analyses summarize at least two independent experiments performed as duplicates and measured in triplicates. Graphs represent the mean value of all measurements plus SEM.

### *CTNNB1* sequencing

For *CTNNB1* sequencing total DNA was extracted using a Maxwell^®^ DNA Purification kit according to manufacturer's instructions (Promega). *CTNNB1* exons were amplified via PCR using the primers listed in Supplementary Table S4. PCR products were precipitated by sodium acetate/ethanol. The resulting DNA pellet was dissolved in 10 μl ddH_2_0, of which 1 μl was applied in the sequencing reaction using the BigDye Terminator v3.1 kit (Applied Biosystems). Sequencing products were purified by precipitating with sodium acetate/ethanol and dissolved in 15 μl highly deionized formamide. Analysis was performed with an ABI3130 Genetic Analyzer (Applied Biosystems).

### Cell culture experiments

The human ARMS cell lines RMS-13 (also known as Rh30) and Rh41 (also called Rh4) and the human ERMS cell line TE671 were obtained from ATCC (for cell lines see [37]). The ERMS and ARMS cell lines were cultured in RPMI, 10% FCS (20% FCS for Rh41), and 1% penicillin/streptomycin.

For generation of stable LEF1 knockdown (LEF1 KD) cell lines, cells were transduced with lentiviral pGIPZ vector (GIPZ Lentiviral shRNAmir Library, Thermo Scientific Open Biosystems) containing LEF1 shRNA TGGAGTTGACATCTGATGG (mature sequence, Thermo Scientific) or empty vector using the packaging cell line HEK293T (ATCC, Rockville, USA). Stable cell lines were selected in medium with puromycin (puromycin dihydrochloride, Sigma-Aldrich Chemistry GmbH). The optimal concentration of puromycin was dependent on the cell line and was 0.5 μg/ml for RMS-13 and 2 μg/ml for Rh41 and TE671. Since the pGIPZ vector expresses GFP, control or shRNA expressing cells could also be visualized and monitored directly by fluorescence. All cell lines were continuously grown in puromycin-containing medium. Puromycin was withdrawn when cells were passaged for the last time before starting an experiment.

For gene expression analysis 300,000 (Rh41, RMS-13, TE671) cells/well were seeded in 6-well-plates. After 24 h, the cells were washed and harvested in TRIzol reagent.

For BrdU incorporation 6,000 cells/well were seeded in 96-well-plates. After 12 h, the cells were incubated for additional 22 h with medium supplemented with 10 μM BrdU. Cell proliferation after BrdU-pulsing was measured using a Cell Proliferation BrdU ELISA (Roche Diagnostics GmbH). BrdU-incorporation is presented as the percentage of the incorporation measured in time-matched vehicle-treated control cells (that was set to 100%).

For Annexin labeling 220,000 cells/well were seeded in 6-well-plates. After 12 h, apoptosis was determined of cells stained with AnnexinV-APC (BD Biosciences) and 7-Amino-Actinomycin D (7-AAD, BD Biosciences). Stained cells were examined by flow cytometry on a LSRII flow cytometer (BD Biosciences), and data were analyzed with the FlowJo software (Tree Star, Inc.).

For cell migration assay 100,000 cells were seeded onto membrane-inserts (translucent track-etched polyethylene terephthalate (PET) membranes with 8 μm pores, BD Biosciences), and incubated for 18 h in a 24-well-plate (BD Biosciences) in 500 μl medium. Simultaneously, 100,000 cells/well were seeded in 24-well-plates in RPMI/10% FCS or RPMI/20% FCS, respectively to measure the cell proliferation. Afterwards the membrane-inserts were transferred into a new 24-well-plate and the cells were stained with 5 μM calcein for 1 h at 37°C. After washing with PBS and removing of cells on top of the membrane (cells which had not migrated), the cells at the bottom of the membrane were analyzed and counted on a microscope (Inverse microscope Carl Zeiss Jena GmbH).

Invasion was measured by assessment of the RMS cell migration rate using an artificial basement membrane in a modified Boyden chamber as described [41]. In short the membrane consisted of a polycarbonate (10 μm pore diameter; Nucleopore) and was coated on ice with Matrigel (ECM gel) diluted 1:4 in serum-free RPMI. 100,000 RMS cells in 500 μl medium were seeded into the upper well of the chamber, while the lower well was filled up to the top with RPMI. 10% FCS served as a chemoattractant. Simultaneously, 100,000 cells/well were seeded in 24-well-plates in RPMI/10% FCS or RPMI/20% FCS to measure the cell proliferation. After 96 h, the floating cells in the lower well were removed, pelleted by centrifugation, resolved in 1 ml PBS and counted.

For TOP/FOP assay 5,000 cells/well were seeded in 96-well-plates. After 24 h, the cells were incubated for additional 48 h with Wnt3a CM. Luciferase activity was measured using the Dual Luciferase Assay Kit (Promega) according to the manufacturer's protocol.

For immunofluorescence staining cells were seeded in 4-chamber culture slides (Thermo Fisher Scientific GmbH) at a density of 40,000 cells/chamber. One day later cells were incubated with Wnt3a CM for 3 h. Immunofluorescence staining was performed as described above.

The data shown summarize two independent experiments performed as triplicates (BrdU incorporation assay, migration assay for RMS-13) or as duplicates (apoptosis assay, migration and invasion assay, TOP/FOP assay). Graphs represent the mean value of all measurements plus SEM.

### Preparation of Wnt3a conditioned medium

Wnt3a conditioned media (Wnt3a CM) or respective control-conditioned media (control CM) were obtained from murine L-cells (ATCC) stably transfected with Wnt3a expression plasmid or from non-transfected L-cells, respectively. Stable murine L-cells that overexpress Wnt3a were maintained in DMEM medium supplemented with 10% FCS, 1% penicillin/streptomycin and 0.4 mg/ml G 418 (G 418 disulfate salt solution, Sigma-Aldrich Chemistry GmbH). Wnt3a CM and control CM were prepared as described by a protocol provided by ATCC [42]. Briefly, the cells (for Wnt3a CM and control CM) were split 1:10 and cultivated with 10 ml fresh medium without G 418 for 4 days. The medium was removed, clarified with a 0.2 μm sterile filter (Omnilab-Krannich) and placed to 4°C. Again, fresh medium (10 ml) was added for another 3 days and processed as described. The first and second batches of conditioned media were pooled and stored at 4°C.

### Transfection of RMS cells

RMS cells were transfected using the NEON Transfection System (Thermo Fisher Scientific) according to the provided protocol. In brief, RMS cells were grown to 70%–90% confluence, harvested and counted. After washing, the cell pellet was resuspended in Resuspension Buffer R (included in the NEON Kit) with a final density of 4,000,000 cells/ml. 400,000 cells were mixed with 5 μg siRNA or 6 μg plasmid DNA in a final volume of 100 μl Buffer R and subjected to electroporation under the following conditions: 1000 V, 2 pulses, pulse time 30 msec. After 48 h the cells were collected by centrifugation at 750 rpm for 5 min and used for subsequent experiments.

Canonical β-catenin-driven WNT signaling activity in RMS cell lines was measured after transfection with *SuperTOPFlash* (TOP) containing multiple TCF/LEF-binding sites or its negative control vector *SuperFOPFlash* (FOP) [43]. *Renilla* reporter plasmid was used for normalization. Wnt3a CM or control CM were added 24 h prior to harvesting. Co-transfection with pCl-neo-β-catS33Y [44] served as positive control.

Knockdown of *β-catenin* expression in RMS cell lines was achieved by using a *β-catenin*-specific siRNA pool (Dharmacon ON-TARGETplus, siRNAs J-003482–09 and J-003482–12) and scrambled siRNA (AllStars negative, Qiagen) was used as control siRNA.

### Western blot analysis

Preparation of cell lysates and determination of protein concentrations were done as described previously [45]. Primary antibodies used to detect the individual target proteins and corresponding secondary antibodies are shown in Supplementary Table S2. All Western blots shown are representative for at least two independent experiments.

### CAM (chorioallantoic membrane) assay

Fertilized White Leghorn chick eggs were incubated at 80% relative humidity and 37.8°C. The eggs were windowed at day 3 and the window was sealed with adhesive tape (Leukosilk, BSN medical). At day 10 of chick development, two million RMS-13 cells/egg were resuspended in 50% RPMI-medium and 50% Matrigel and incubated for 30 min at 37°C, 5% CO_2_ before applying them on the CAM. The tumors were dissected after 7 days (day 17 of chick development), fixed in 4% paraformaldehyde for 20 min, washed twice in PBS and transferred into 10% sucrose for 3 h at 4°C and 30% sucrose ON at 4°C. Tumors were then embedded in tissue freezing medium and cut with a cryotome into 14 μm thick sections. The experiments were performed according to the guidelines of the European Parliament (2010/63/EU) and the council for the protection of animals in science (§14 TierSchVersV). Tumors and tumor cells were visualized by intravital GFP imaging and immunofluorescence.

### Statistical analyses

If not otherwise indicated, statistical differences were analyzed using Mann-Whitney testing or Student's *t*-test. Data was considered significant when *P* < 0.05.

## SUPPLEMENTARY MATERIALS TABLES AND FIGURES


