# Supplementary material for: LEF1 reduces tumor progression and induces myodifferentiation in a subset of rhabdomyosarcoma
Source: Oncotarget. 2016 Dec 10;8(2):3259–73. doi: 10.18632/oncotarget.13887 (PMC5356880; doi:10.18632/oncotarget.13887)
Supplement: Supplementary file 1 [file oncotarget-08-3259-s001.pdf]

LEF1 reduces tumor progression and induces myodifferentiation in a subset of rhabdomyosarcoma

Supplementary Materials

Supplementary Table S1: The expression of *LEF1* and *CTNNB1* was evaluated in a publicly available RMS microarray data set [1]

	LEF1	CTNNB1
**ARMS (P3F) (*n* = 38) vs. ERMS (*n* = 75)**	0.732	0.947
**ARMS (P3F) (*n* = 38) vs. ERMS (w.d.) (*n* = 21)**	0.360	0.715
**ARMS (P3F) (*n* = 38) vs. ERMS (m.d.) (*n* = 33)**	0.997	0.499
**ARMS (P3F) (*n* = 38) vs. ERMS (u.d.) (*n* = 21)**	0.986	0.176

Shown are the adjusted *P*-values of the comparison of *LEF1* and *CTNNB1* expression in RMS subgroups. P3F: PAX3-FOXO1-fusion positive; w.d. well differentiated; m.d. moderately differentiated; u.d. undifferentiated.

Supplementary Table S2: Primary and secondary antibodies used for immunohistochemical analyses

Antibody	Product and company	Dilution
**Primary antibody**		
Mouse anti-β-catenin	pAB, 610153, BD Bioscience	1:250
Mouse anti-β-catenin	mAB, CAT-5H10, Zymed	1:200
Mouse anti-HLA-A,B,C	mAB, 311402, BioLegend	1:100
Rabbit anti-LEF1	mAB, EPR2029Y, Abgent	1:250
**Secondary antibody**		
Rhodamine (TRITC) Donkey anti-Mouse IgG (H+L)	pAB, 715-025-150, Jackson ImmunoResearch	1:200
Alexa Fluor 594 Goat anti-Mouse IgG2a (γ2a)	pAB, A-21135, ThermoFisher	1:200
EnVision Detection Systems Peroxidase/DAB Rabbit/Mouse	pAB, K4065, DAKO	Manufacturer instructions

Primary and secondary antibodies used for Western blot

Antibody	Product and company	Dilution
**Primary antibody**		
Mouse anti-β-catenin	pAB, 610153, BD Bioscience	1:10000
Mouse anti-HSC-70	mAB, sc-7298, Santa Cruz	1:10000
Rabbit anti-β-Actin	mAB, 13E5, Cell Signaling	1:10000
Mouse anti-β-Actin	mAB, C4, Santa Cruz	1:2000
Rabbit anti-LEF1	mAB, EPR2029Y, Abgent	1:20000
Rabbit anti-LEF1	mAB, C18A7, Cell Signaling	1:1000
**Secondary antibody**		
Goat anti-Rabbit/HRP	pAB, A0545, Sigma-Aldrich	1:5000
Sheep anti-Mouse/HRP	pAB, NA931, GE Healthcare	1:5000

Supplementary Table S3: Oligonucleotides used for qRT-PCR

Primer name	Primer sequence (5′ - 3′ orientation)	Location	Application
18S_For	CGCAAATTACCCACTCCCG	exon 1	*18S* expression
18S_Rev	TTCCAATTACAGGGCCTCGAA	exon 1	
AXIN2_For	GCCAACGACAGTGAGATATCC	exon 2	*AXIN2* expression
AXIN2_Rev	CTCGAGATCAGCTCAGCTGCA	exon 4	
CKM_For	GGAACTCTTTGACCCCATCA	exon 3	*CKM* expression
CKM_Rev	CTCCACCCTTGAGGTTTTCA	exon 3/4	
CTNNB_For	GAAACGGCTTTCAGTTGAGC	exon 8	*CTNNB1* expression
CTNNB_Rev	CTGGCCATATCCACCAGAGT	exon 9/10	
c-MYC_For	GTGCTCCATGAGGAGACA	exon 2	*c-MYC* expression
c-MYC_Rev	AGCCTGCCTCTTTTCCA	exon 3	
DESMIN_For	TGAAGGGCACTAACGATTCC	exon 5/6	*DESMIN* expression
DESMIN_Rev	TGTTGTCCTGGTAGCCACTG	exon 6	
GAPDH_For	TGCACCACCAACTGCTTAGC	exon 5	*GAPDH* expression
GAPDH_Rev	GGCATGGACTGTGGTCATGAG	exon 5/6	
LEF1_For	CGGGTACATAATGATGCCAA	exon 3	*LEF1* expression
LEF1_Rev	CGTCACTGTAAGTGATGAGGG	exon 4	
MYH1_For	TGTGCAGCAGGTGTACAATGC	exon 13/14	*MYH1* expression
MYH1_Rev	TGCACAGCTGCTCCAGGCT	exon 15	
MYOD_For	CGAACCCCAACCCGATA	exon 3	*MYOD* expression
MYOD_Rev	GAAAAAACCGCGCTGTGT	exon 3	
MYOGENIN_For	AGCGAATGCAGCTCTCACAG	exon 2	*MYOGENIN* expression
MYOGENIN_Rev	AGGTTGTGGGCATCTGTAGG	exon 4	
TCF1_For	GCAACCTGAAGACACAAGCA	exon 4/5	*TCF1* expression
TCF1_Rev	GCAATGACCTTGGCTCTCAT	exon 5	
TCF3_For	GAGTCGGAGAACCAGAGCAG	exon 1	*TCF3* expression
TCF3_Rev	CTGTCCTGAGGCCTTCTCAC	exon 2/3	
TCF4_For	ATGCTTCCATGTCCAGGTTC	exon 8/9	*TCF4* expression
TCF4_Rev	CACTCTGGGACGATTCCTGT	exon 9	

Supplementary Table S4: Oligonucleotides used for *CTNNB1* sequencing

Primer name	Primer sequence (5′ - 3′ orientation)	Position	Product size
CTNNB_Sq_1F	ACAGGTATCCCAGTGACTTAGG	−124 bp	182 bp
CTNNB_Sq_1R	GAAGCAGAGCCCCAATTCAG	intron 1	
CTNNB_Sq_F2	TCTGCTTTTCTTGGCTGTCT	intron 1	474 bp
CTNNB_Sq_R2	ACTCTCTTTTCTTCACCACAACA	intron 2	
CTNNB_Sq_F3	TGCTGAACTGTGGATAGTGAGT	intron 2	659 bp
CTNNB_Sq_R3	AAGCATTTTCACCAGGGCAG	intron 4	
CTNNB_Sq_F4	CTGCCCTGGTGAAAATGCTT	intron 4	358 bp
CTNNB_Sq_R4	AGGTGTCCAATGCTCCATGA	intron 5	
CTNNB_Sq_F5	TTAGGTCCAATGGCAAGCTG	intron 5	392 bp
CTNNB_Sq_R5	GGCTGCAAACTGAATAGGACC	intron 6	
CTNNB_Sq_F6	ACTTCTAGCTAATGACTAGGGCC	intron 6	716 bp
CTNNB_Sq_R6	TACCCCTATCGCAGCCATAC	intron 8	
CTNNB_Sq_F7	GTATGGCTGCGATAGGGGTA	intron 8	416 bp
CTNNB_Sq_R7	TTCAGGAAGACGGATGGGG	intron 9	
CTNNB_Sq_F8	GTTACGGGGAACTTCGGGTA	intron 9	403 bp
CTNNB_Sq_R8	CCTCCCTCTTCTCAAGTCTCA	intron 10	
CTNNB_Sq_F9	TTGGGAATGTTTGCACCACA	intron 10	558 bp
CTNNB_Sq_R9	GCCAGGGAAACATCAATGCA	intron 12	
CTNNB_Sq_F10	TGAGGTGTGGAGTTTTGAAGA	intron 12	415 bp
CTNNB_Sq_R10	TGTGTGGCTCTGAATTCCCT	intron 13	
CTNNB_Sq_F11	GGATGCCCTAACCTCAGTGT	intron 13	401 bp
CTNNB_Sq_R11	CCACCCTACCAACCAAGTCT	3′ UTR	

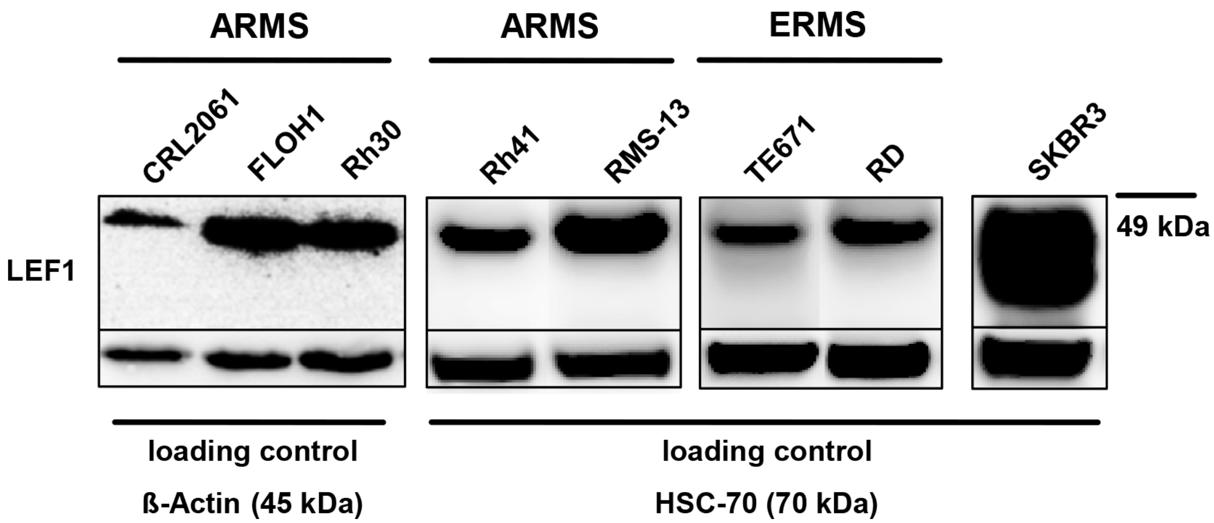

Supplementary Figure S1: LEF1 protein levels analyzed by Western Blot in the ARMS cell lines CRL2061, FLOH1, Rh30, Rh41 and RMS-13 and in the ERMS cell lines TE671 and RD. The cell line SKBR3 served as a positive control. β-Actin or HSC-70 protein levels served as loading control.

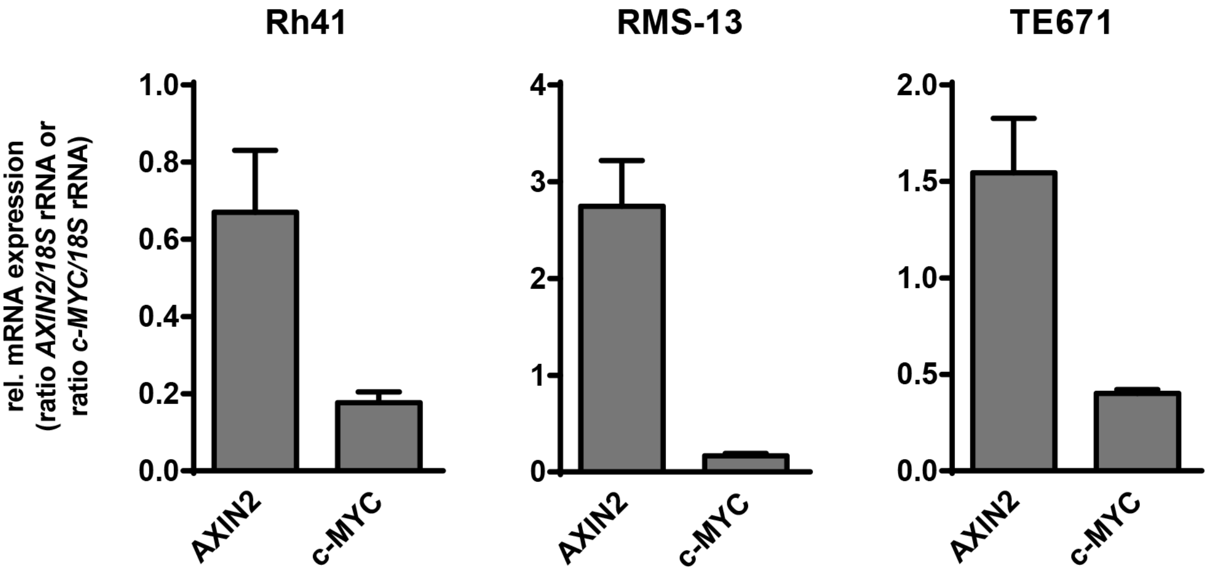

Supplementary Figure S2: *AXIN2* and *c-MYC* expression levels analyzed by qRT-PCR in Rh41, RMS-13 and TE671 wildtype cells. Gene expression levels were normalized to *18S* rRNA expression levels. All data represent mean+SEM of three independent experiments performed in duplicates and measured in triplicates.

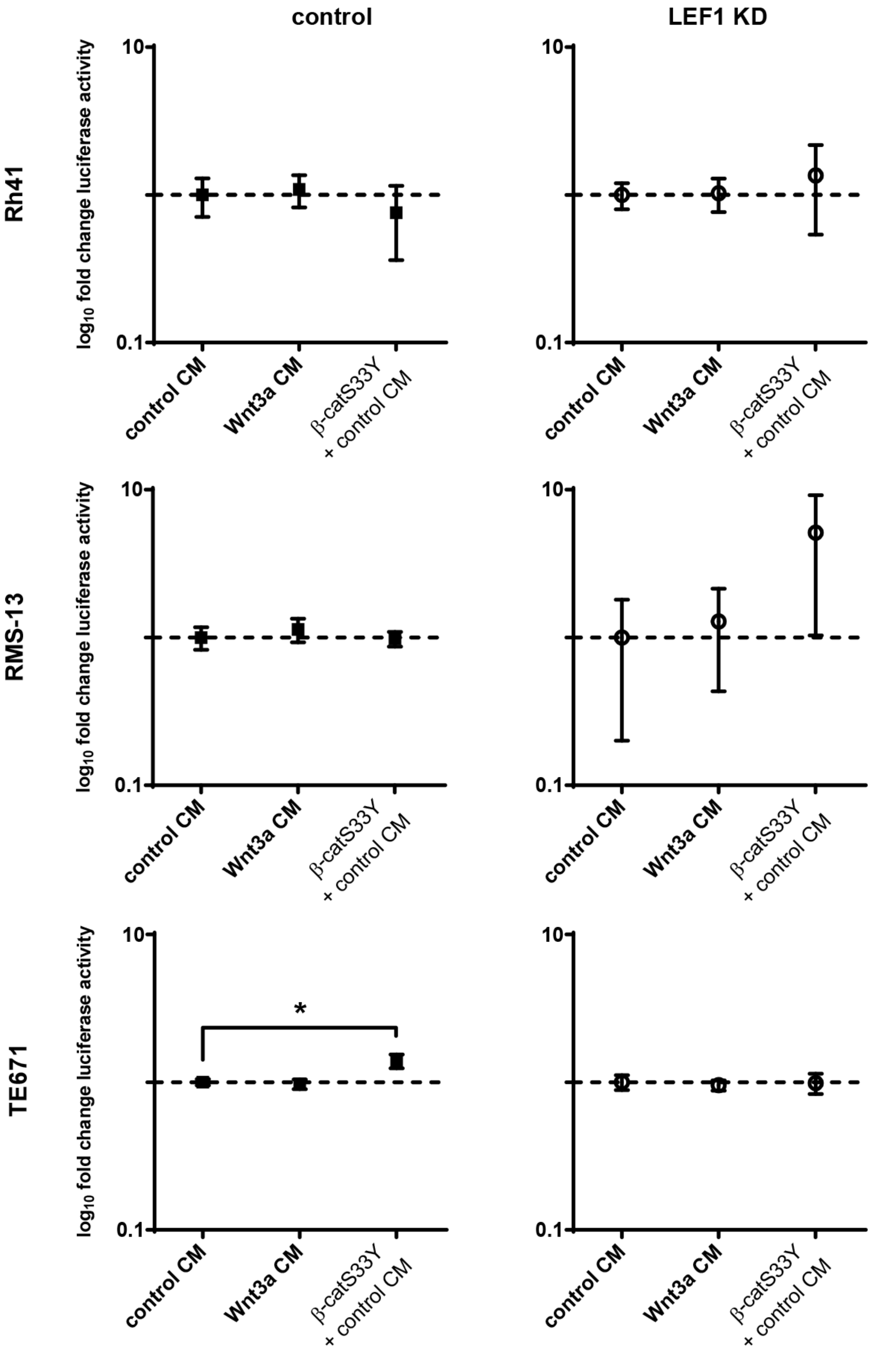

Supplementary Figure S3: The stable RMS LEF1 KD and respective control cells were transfected with the *SuperFOPFlash* (FOP) plasmid along with *Renilla* reporter plasmid for normalization. The FOP assay was performed in the presence of Wnt3a conditioned medium (Wnt3a CM) that activate canonical WNT signaling, or in the presence of control medium (control CM) or after transfection with pCl-neo-β-catS33Y plasmid that expresses an activated β-catenin. The value after incubation with control CM was set to 1. As demonstrated, with the exception of pCl-neo-β-catS33Y-transfected TE671 cells ($P = 0.041$), all other settings had no effect on luciferase activity in FOP-transfected RMS cell lines.

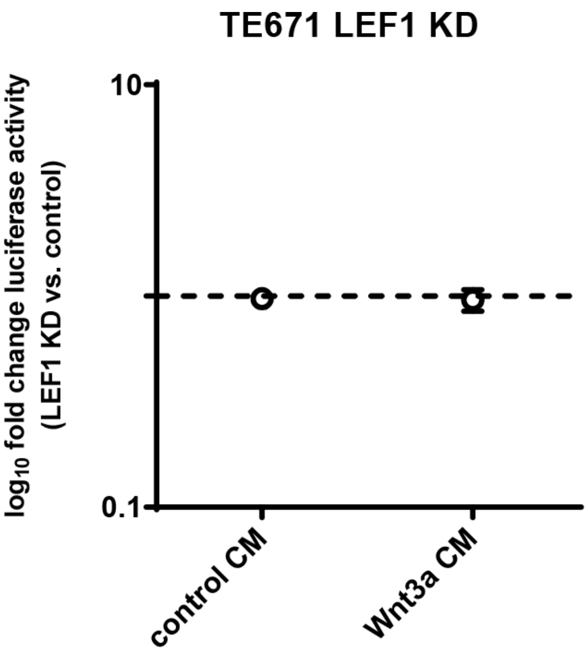

Supplementary Figure S4: As stated in the main manuscript, both TE671 control and TE671 LEF1 KD cells showed a more than 10-fold induction of TOP activity after Wnt3a CM treatment (Figure 3B). In order to evaluate to what extend the LEF1 KD influences Wnt3a-mediated TOP induction, the values from the TOP assay of TE671 LEF1 KD cells were normalized to that of TE671 control cells (that were set to 1). As shown below, the LEF1 KD in TE671 did not influence reporter activity when compared to control-transduced cells i.e. we did not see any increase or decrease in luciferase activity that could be ascribed to the LEF1 KD.

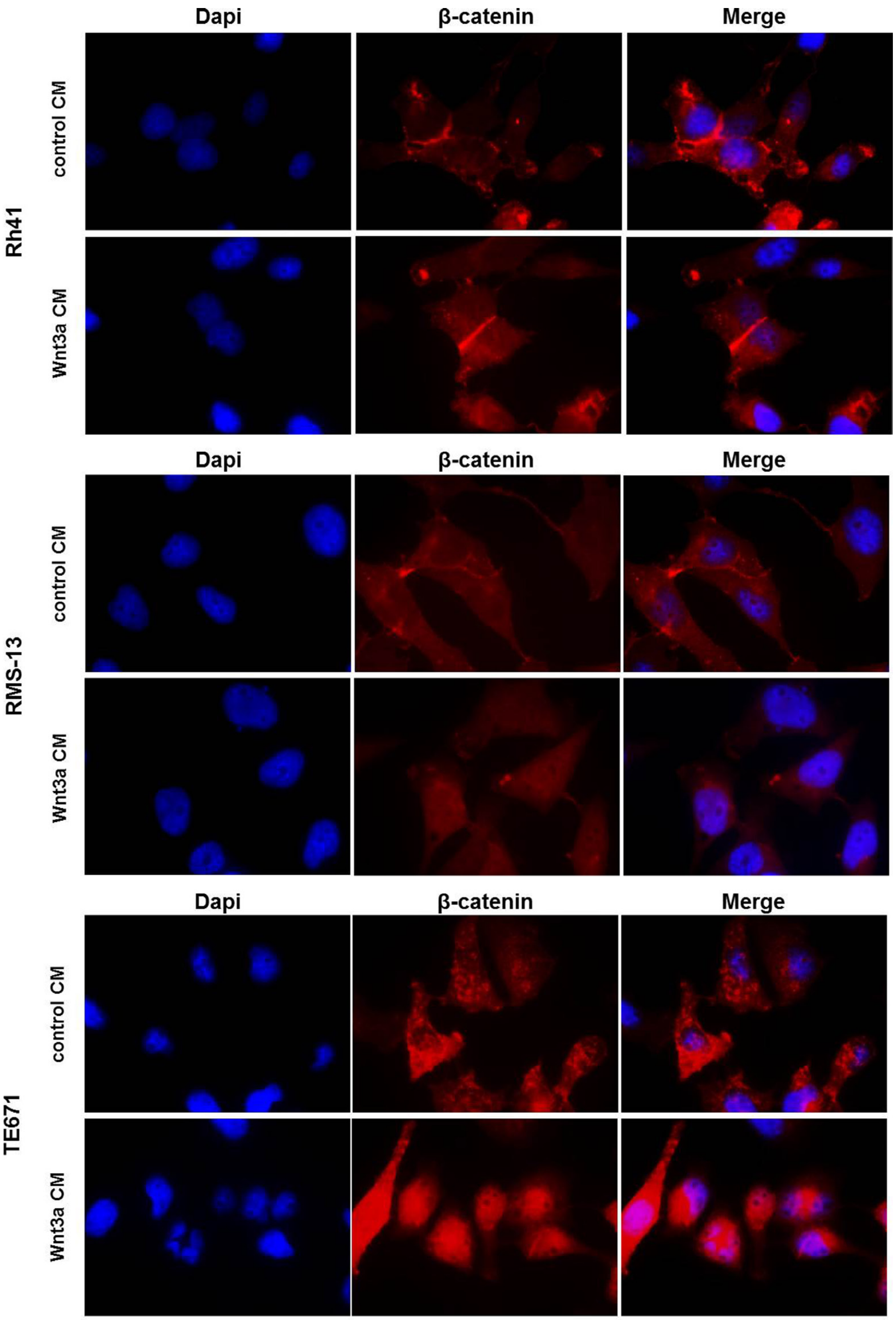

Supplementary Figure S5: Localization of β-catenin in Rh41, RMS-13 and TE671 control transduced cell lines cultured in control or Wnt3a CM for 3 h as analyzed by immunofluorescence (60-fold magnification). Approximately 10% of Wnt3a-treated Rh41 cells showed weak nuclear signals, but none of Wnt3a-treated RMS-13 cells. In TE671 cells, staining revealed a predominant nuclear localization of β-catenin after Wnt3a stimulation. DAPI was used to stain cell nuclei (blue).

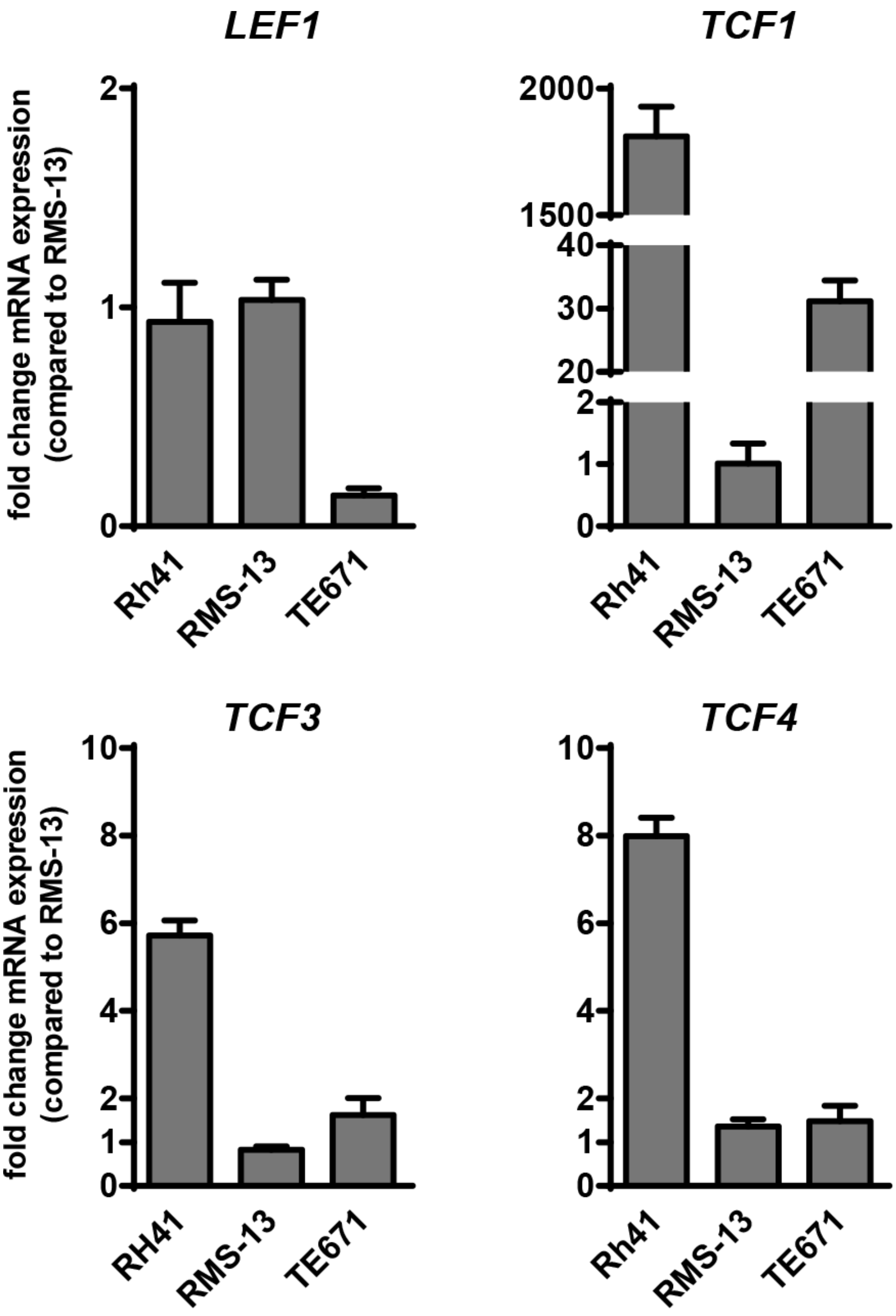

Supplementary Figure S6: Expression of *LEF1*, *TCF1, TCF3* and *TCF4* in Rh41, RMS-13 and TE671 are shown as fold change expression in relation to RMS-13 cells. Gene expression levels were normalized to *GAPDH* expression levels. Data represent mean + SEM of at least four independent experiments measured in duplicates. Please note that the expression level of one of the independent measurements of RMS-13 cDNA was set to 1.

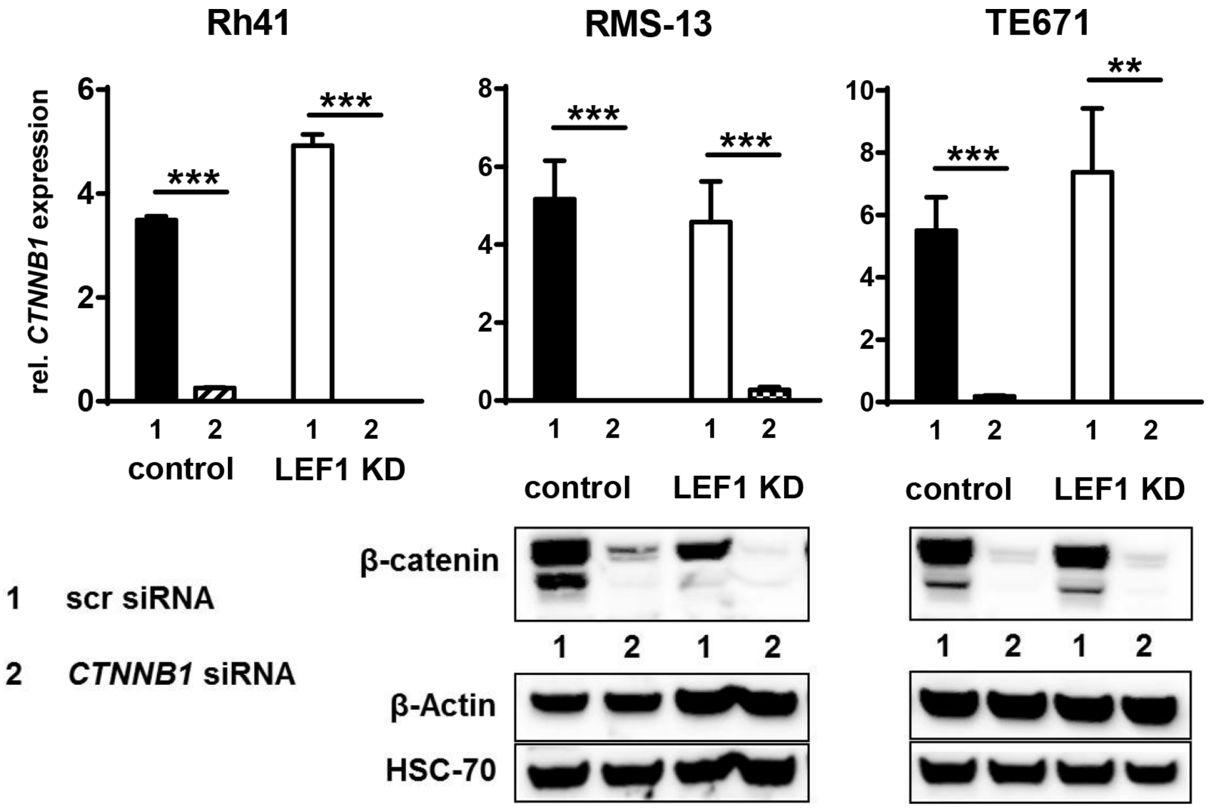

Supplementary Figure S7: qRT-PCR and Western blot analysis of β-catenin in Rh41, RMS-13 and TE671 control and the respective LEF1 KD cells performed 72 h (qRT-PCR) and 96 h (Western blot) after transfection with scrambled (scr) siRNA (1) or *CTNNB1* siRNA (2). qRT-PCR (upper panel) data represent two independent experiments performed in duplicates and measured in triplicates. Gene expression levels were normalized to *18S* rRNA and data are displayed as mean+SEM; $^{**}P < 0.01$, $^{***}P < 0.001$ by Students *t*-test. For Western blot (lower panel) analysis was done for RMS-13 and TE671 cells. HSC-70 and β-Actin served as loading control.

REFERENCES

1. Davicioni E, Anderson MJ, Finckenstein FG, Lynch JC, Qualman SJ, Shimada H, Schofield DE, Buckley JD, Meyer WH, Sorensen PH, Triche TJ. Molecular classification of rhabdomyosarcoma—genotypic and phenotypic determinants of diagnosis: a report from the Children's Oncology Group. Am J Pathol. 2009; 174: 550–564.
